# Evaluation and comparison of electromyographic activity in bench press with feet on the ground and active hip flexion

**DOI:** 10.1371/journal.pone.0218209

**Published:** 2019-06-14

**Authors:** José M. Muyor, David Rodríguez-Ridao, Isabel Martín-Fuentes, José A. Antequera-Vique

**Affiliations:** 1 Laboratory of Kinesiology, Biomechanics and Ergonomics (KIBIOMER Lab.), Research Central Services, University of Almería, Almería, Spain; 2 Health Research Centre, University of Almería, Almería, Spain; 3 Faculty of Education Sciences, Universidad de Almería, Almería, Spain; São Paulo State University (UNESP), BRAZIL

## Abstract

The present study aimed to evaluate and compare the levels of electromyographic activation in the pectoralis major, anterior deltoid, triceps brachii, forearm, rectus abdominis, external oblique, and rectus femoris muscles during a horizontal bench press in two situations: 1) with the feet on the ground; and 2) with active hip flexion and 90° of knee flexion. Twenty young men were familiarized with the procedure and the calculation of one-rep max (1RM). In a second session, electromyographic activity values were recorded in both bench press situations (with the feet on the ground vs active hip flexion and knees at 90°) at 60% 1RM. Performing the bench press with the hips and knees flexed produced significantly greater muscle activation of all elevated muscles (*p* < 0.01; *d* > 0.5). The pectoralis major showed the greatest activation, followed by the anterior deltoid and the triceps brachii. In addition, the greater activation of the abdominal muscles occurs due to the need to stabilize the core while performing the bench press with hip and knee flexion as well as the lumbar spine due to traction of the hip flexors.

## Introduction

The bench press is one of the most widely used exercises in sports training to condition the muscles and evaluate upper body strength in both novice [1] and advanced [2–4] athletes. It is also one of the basic exercises in powerlifting competitions [5]. For this reason, scientists and strength and conditioning coaches have developed interest in the bench press characteristics that are associated with maximum strength, explosive strength, and power as well as the level of muscle activation as a function of variations in its methodology [6]. In this sense, strength and conditioning coaches often change the body position in the bench press exercise to modify the implications for the activation of certain muscles or increase safety during execution [7]. As indicated by Trebs et al. [8], knowing the muscle activation level or modification by variation in posture or exercise is a fundamental key to developing strength and muscle mass.

This has resulted in studies evaluating the level of muscle activation in the bench press according to psychological variables, such as, attentional focus [9, 10] or verbal instruction provided during the exercise [11]. Other studies have analyzed the muscle activation in this exercise according to biomechanical variables, such as the kinematics of the movement [12], the width of the grip bar and the bench’s inclination level [7, 8]. Other studies have compared the muscle activation level of the bench press with other exercises, machines, and/or equipment for muscle conditioning [13–15]. Finally, other studies have evaluated the muscle activation according to the stability/instability of the surface when the bench press is executed [16–20]. Although, all of them have focused on the instability caused on the supporting surface of the body, using a balance cushion [16, 20] or a Swiss ball [16, 17, 19]. Only, Norwood et al. [18] generated instability in the lower limbs by placing their feet on a Bosu. However, these authors only evaluated the electromyography activity of the trunk stabilizers, and not agonist muscles such as pectoralis major, anterior deltoid, triceps brachii or forearm muscles.

Concretely, the bench press exercise has three points of support that give stability: the body, which is lying supine on the bench, and each foot (right and left), both resting on the ground. In this line, one modification frequently used in the bench press exercise involves performing it with active hip and knee flexion at 90° [21]. Normally, strength and conditioning coaches indicate that this technique “protects” the spinal column, by being completely supported on the horizontal bench, from loss of the lumbar lordosis due to the hip flexion and consequent pelvic retroversion. However, no studies to date have evaluated the level of muscle activation in this position.

The objective of the present study was to evaluate and compare the levels of electromyographic (EMG) activation of the pectoralis major (clavicular portion, sternal portion, and costal portion), anterior deltoid, triceps brachii (medial head), forearm (flexor digitorum), rectus abdominis, external oblique, and rectus femoris (quadriceps) in the horizontal bench press under these conditions: 1) with the feet on the ground, maintaining the hips in a neutral position; and 2) with active hip flexion and 90° of knee flexion.

## Materials and methods

### Participants

A total of 20 young healthy physically active adults with an experience of 4 years in strength training, specifically the bench press, voluntarily participated in the present study. The characteristics of the sample are shown in Table 1.

**Table 1:** Descriptive characteristics of sample. Mean (standard deviation).

	Mean (SD)
Age (years)	22.80 (3.00)
Body mass (kg)	77.00 (8.88)
Height (m)	1.79 (0.05)
BMI (kg·m^-2^)	23.83 (2.16)
150% biacromial distance (cm)	69.52 (3.99)
1RM Bench press (kg)	85.00 (12.87)

The participants were required to be free of any injury or discomfort that would impede their performance of the exercises in the study and not have sustained a musculoskeletal injury in the 12 months prior to the study. The participants were also asked not to ingest stimulants or perform any vigorous exercise during the 24 hours before the study. Failure to comply with any of the instructions above resulted in elimination from the study sample.

Prior to the evaluation, all participants were informed verbally and in writing of the study objectives and procedures, which were previously designed according to the Declaration of Helsinki and approved by the Ethics and Research Committee of the University of Almería (Spain). Moreover, the individual in this manuscript has given written informed consent (as outlined in PLOS consent form) to publish these case details. All study participants voluntarily signed an informed consent form.

### Procedures

All participants visited the Fitness Centre two times during the five-week study period. These visits were separated with a minimum of 2 days to avoid muscle fatigue effects [20]. The first session was used to familiarize the participants and determine the load lifted in a maximum repetition (1RM) in the bench press exercise, while the second test session was used for electromyographic recording of the muscles to evaluate the bench press in two situations: 1) with the feet on the ground and the hips in a neutral position on the bench; and 2) with the hips and knees flexed at 90°. These both conditions were performed in a randomized order.

#### Session 1: Familiarization with the bench press exercises and determination of 1RM

This session began by determining each participant’s height using a Seca stadiometer (Seca, Hamburg, Germany), body mass using an electronic weigher (model BF-350; Tanita, Tokyo, Japan), and biacromial distance (Table 1).

Before 1RM was determined, each participant performed a warm up consisting of a 10-minute cardiovascular exercise on an elliptical machine (to mobilize the upper extremities) at 40–60% of the maximum heart rate reserve followed by a 5-minute session of dynamic-active stretching and joint mobility exercises in the body segments involved in the bench press exercise. Next, the participants performed a specific warm up protocol on the bench press to reach the 1RM [12, 22]. They performed 4 warm up sets: 1) twenty repetitions approximately at 30% of 1RM, 2) twelve repetitions approximately at 50% of 1RM, 3) six repetitions approximately at 70% of 1RM and 4) one repetition approximately at 85% 1RM. Finally, the participants had to lift, maintaining adequate technique, a single repetition of maximum weight possible that was considered as 1RM [23]. In this line, were done a maximum of three attempts to establish 1RM. The rest periods between sets were around 3 to 5 minutes to avoid possible fatigue.

After the 1RM value was determined, the participants were familiarized with the technique described by Padulo et al. [24] for both bench press exercises (with the feet on the ground and with the hips and knees flexed at 90°). The participants practiced both exercises at 40% 1RM at least three times each until the investigators were satisfied with the technique and the participants felt comfortable and secure with the technical execution of both [10].

#### Session 2: Electromyography setup and data collection

Participants were asked to refrain from any lifting or exertion with the upper body at least 48 hours before this test period [8]. The protocol began with preparation of the participants’ skin by shaving the hair off the body areas where the electrodes would be placed for electromyography. After this, each participant completed the same cardiovascular warm up, and dynamic-active stretching and joint mobility exercises as in session 1.

Next, the skin was cleaned with 96% alcohol and cotton. Bipolar adhesive Ag/AgCl electrodes (Medico Lead-Lok, Noida, India) were then placed parallel to the muscle fibers at 2-cm intervals with the reference electrode far from the electrode pair in accordance with the manufacturer’s specifications.

**Fig 1:**
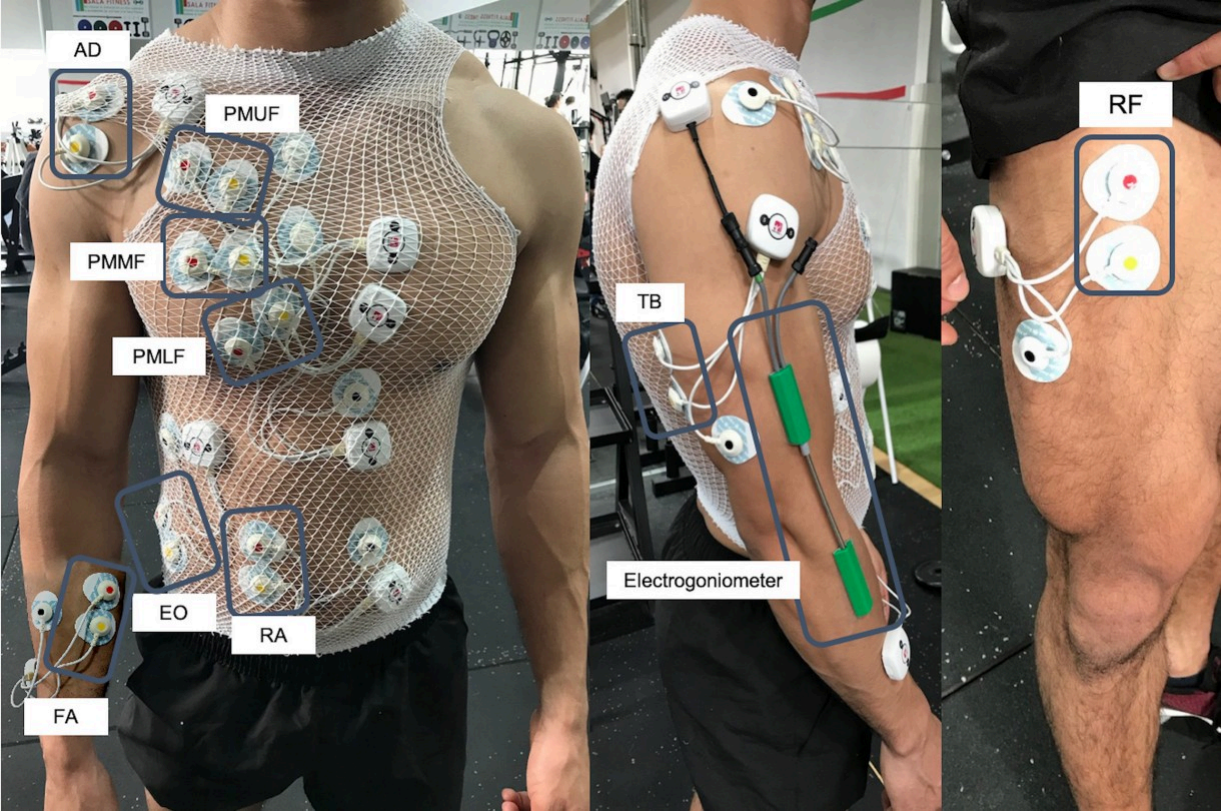
Electrode and electrogoniometer placement diagram for recording sEMG signals and elbow movement in each series. AD: Anterior deltoid; PMUF: Pectoralis major upper fibers; PMMF: Pectoralis major middle fibers; PMLF: Pectoralis major lower fibers; RA: Rectus abdominis; EO: External obliques; FA: Forearm; TB: Triceps brachii; RF: Rectus femoris.

The electrodes were placed on each participant’s dominant side on the pectoralis major upper fibers (clavicular portion -PMUF) on the midclavicular line over the second intercostal space [25]; pectoralis major middle fibers (sternal portion -PMMF) on the chest wall horizontal from the arising muscle mass (approximately 2cm out from the axillary fold) [26]; pectoralis major lower fibers (costal portion -PMLF) on the midclavicular line over the fifth intercostal space [25]; anterior deltoid (AD) on 1.5 cm distal and anterior to the acromion [16]; triceps brachii, medial head (TB) at the midpoint between the posterior aspect of the acromion and the olecranon processes [27]; forearm (flexor digitorum -FA) in the middle of the muscle belly between the joints of the wrist and the elbow [28]; rectus abdominis (RA) on the 3cm lateral to the midline and midway between the xiphoid process and the umbilicus [29]; external oblique (EO) superior to the anterior superior iliac spine at an oblique angle, at the level of the umbilicus [29]; and rectus femoris, quadriceps (RF) at a point in midway between the anterior iliac spine and the superior part on the patella [30]. All electrodes were placed in accordance with the Surface Electromyography for the Non-invasive Assessment of Muscles (SENIAM) recommendations [31]. All electrodes were covered with a bandage to prevent possible displacement during the execution of the bench press exercise (Fig 1).

### Data collection

The maximal voluntary isometric contraction (MVIC) of each muscle was then recorded to normalize the EMG values that would be registered in the bench press exercise. To do so, two maximum isometric contractions were performed for 3 seconds with a 10-sec rest between contractions [32] and 2 minutes between the MVIC evaluation of each muscle [33]. Specifically, the MVIC were performed as follows: For the Anterior deltoid participants performed a deltoid flexion at 90° in a seated position an erect posture with no back support. For the Pectoralis major (upper, middle and lower portions), participants performed a bench press with a grip at 150% of biacromial width, the shoulder abducted at 45° and feet flat on the bench. For Rectus abdominis, participants were positioned supine in a hook-lying position with the feet supported and the thoracolumbar spine maximally flexed (curl-up position). Manual resistance was applied to the subject´s shoulders in the direction of trunk extension. For External obliques, participants were supine in a hook-lying position with the feet flat on the support surface. The trunk was maximally flexed and rotated to the left, with manual resistance at the shoulders applied in the direction of trunk extension and right rotation. For the Rectus femoris, participants were supine on the table. The action of the Rectus femoris was tested across the hip and knee simultaneously. The subject´s right hip was flexed to 45° with knee extended. Resistance was applied by the tester on the distal leg just proximal to the malleoli. For each participant, an examiner provided verbal encouragement to maintain a consistent effort during the MVIC. After each MVIC, each subject was queried if he or she believed the effort was a maximum effort. If not, the MVIC was repeated after 2 minutes rest [33]. The intraclass correlation coefficients (ICCs) ≥ 0.98; and the coefficient of variation (CV) ≤ 3% demonstrated high reliability in all MVIC evaluated.

**Fig 2:**
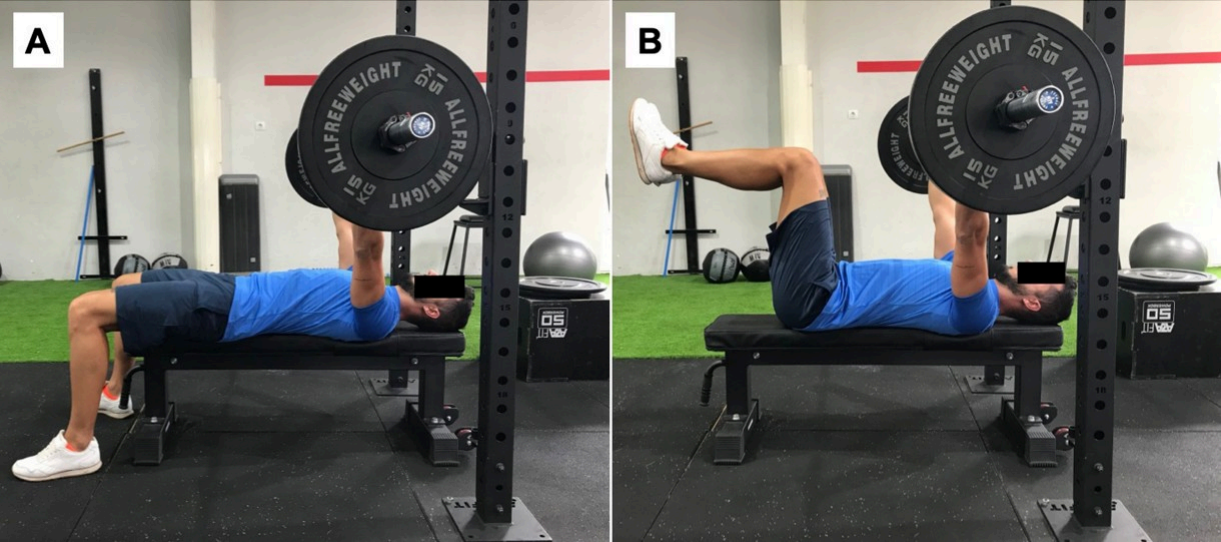
A. Bench press exercise with the feet on the ground, knees flexed at 90°, and pelvis and lumbar zone maintained in a neutral position. B. Bench press exercise with hip and knee flexed at 90° without resting the feet on the ground.

Next, participants performed a more specific warm up on the bench press with 20 repetitions at 30% 1RM. Finally, after 5 minutes rest, began the data collection on the bench press in both situations: 1) with the feet on the ground, knees flexed at 90°, and pelvis and lumbar zone maintained in a neutral position (Fig 2A); and 2) with hip and knee flexed at 90° without resting the feet on the ground (Fig 2B). For each bench press exercise, participants completed a set of 8 repetitions at 60% 1RM, at a rate of 2:2 (two times for the eccentric phase and two times for the concentric phase) [34, 35], controlled by a KORG MA-1 metronome (Keio Electronic Laboratories, Tokyo, Japan) at 60 beats per minute. Both exercises were evaluated randomly with a 10-min break allowed between executions. The ICCs were ≥ 0.95 and CV < 3% in the EMG in all muscles evaluated on the bench press in both situations examined.

Regarding the bench press exercise, all participants started the exercise lying in the supine position on the bench gripping the bar (using a *hook* grip with the thumb) with a width of 150% of the width corresponding to the biacromial distance and the hands and forearms in pronation during all repetitions. In the eccentric phase of the exercises, the bar was lowered to 1 cm from the chest (sternum) with the shoulders abducted to approximately 45°. In the concentric phase, the bar was raised until the elbows were extended (avoiding elbow hyperextension) to stay in the start position. The participant’s head and back (thoracic area) were kept in contact with the bench to prevent any inertial motion and the bar was kept completely perpendicular to the sternum (at the height of the nipples).

Following Nascimento et al. [20] the load selected for the electromyographic analysis of the bench press exercise in both situations (with the feet supported and unsupported) was performed at 60% of 1RM. This relative load of 60% 1RM was chosen because it is the lowest recommended load for strength training [36, 37] and coincides with Marshall and Murphy [17] for reasons of participant safety due to having to perform a strength exercise in an unstable situation (with no support from the feet).

### Electromyography

The EMG signal of the evaluated muscles was recorded by a WBA Mega device (Mega Electronics, Ltd., Kuopio, Finland) at a sampling frequency of 1000 Hz. The analog signal was converted to digital via an A/D converter (National Instruments, New South Wales, Australia) and filtered by bandwidth (12–450 Hz) with a fourth-order Butterworth filter through the software program LabView (National Instruments, Austin, TX, USA). The raw EMG signals were then converted into root-mean-square (RMS) signals with the software program MEGAWIN (Mega Electronics, Ltd.) for further analysis later.

To limit the effects of fatigue, the bench press exercise was performed 10 minutes after the MVIC evaluations were performed [38].

To help identify the different repetitions during the EMG signal analysis, the angle of the elbow was continuously recorded with an electrogoniometer (Biometrics Ltd., Newport, UK) placed on the lateral side of the ulna and the humerus (Fig 1). The data from the electrogoniometer were continuously recorded and synchronized with the EMG data using EMG equipment (Mega Electronics, Ltd.).

### Statistical analyses

First, the data distribution was analyzed by the Shapiro-Wilk normality test. As all variables followed a normal distribution, the different statistical analyses were performed based on the parametric tests.

The results were analyzed by a statistical description of each of the dependent variables to obtain the mean values and standard deviations. Relative reliability of the measurements was calculated by the ICC with 95% confidence interval, using the one-way random effects model, whereas absolute reliability was assessed using the CV.

Student's *t*-test for paired samples was performed to compare the EMG values expressed in mV as well as in % of MVIC in the two bench press exercises.

The effect size was calculated through Cohen's *d* using the combined standard deviation formula [39]. An effect size *d* > 0.8 was considered large, while *d* at approximately 0.5 was considered moderate and *d* < 0.2 was considered small [39]. The statistical power and effect sizes were calculated with the software program G*power 3.1 for Mac OS X [40]. The statistical power was > 0.9 for all of the variables analyzed with the sample size used in the present study. Statistical data were analyzed with the software program IBM SPSS (v. 25) with a level of significance of *p* < 0.05.

## Results

The bench press exercise with the hips and knees flexed at 90° showed significantly greater muscle activation of all evaluated muscles (*p* ≤ 0.01; *d* > 0.5) (Table 2).

**Table 2:** Muscle activity (expressed as mV) of the pectoralis major (upper, middle, and lower fibers), anterior deltoid, triceps brachii, forearm, rectus abdominis, external oblique, and rectus femoris during bench press with the feet on the ground vs with the feet suspended and hips flexed.

	Bench press with the feet on the ground	Bench press with the suspended feet and flexed hips	Mean difference (95% CI)	*p*-value	Effect size (*d*)
Pectoralis major upper fibres (clavicular portion) (mV)	328.00 ± 119.01	378.35 ± 124.60	50.35 ± 39.49	< 0.001	1.26
Pectoralis major middle fibres (sternal portion) (mV)	308.55 ± 200.78	335.95 ± 197.51	27.40 ± 32.31	0.001	0.84
Pectoralis major lower fibres (costal portion) (mV)	335.20 ± 152.37	371.65 ± 148.89	36.45 ± 38.40	< 0.001	0.93
Anterior deltoid (mV)	565.65 ± 247.69	622.20 ± 288.69	56.55 ± 100.86	0.021	0.56
Triceps brachii (medial head) (mV)	240.10 ± 88.46	267.30 ± 88.63	27.20 ± 24.80	< 0.001	1.09
Forearm (flexor digitorum) (mV)	144.15 ± 97.06	163.25 ± 114.54	19.10 ± 30.92	0.012	0.61
Rectus abdominis (mV)	31.35 ± 11.15	70.80 ± 42.98	39.45 ± 43.25	0.001	0.91
External oblique (mV)	17.50 ± 9.20	70.85 ± 57.74	53.35 ± 53.89	< 0.001	0.98
Rectus femoris (quadriceps) (mV)	10.47 ± 3.61	81.52 ± 40.11	71.05 ± 41.86	< 0.001	1.69

CI: confidence interval.

**Fig 3:**
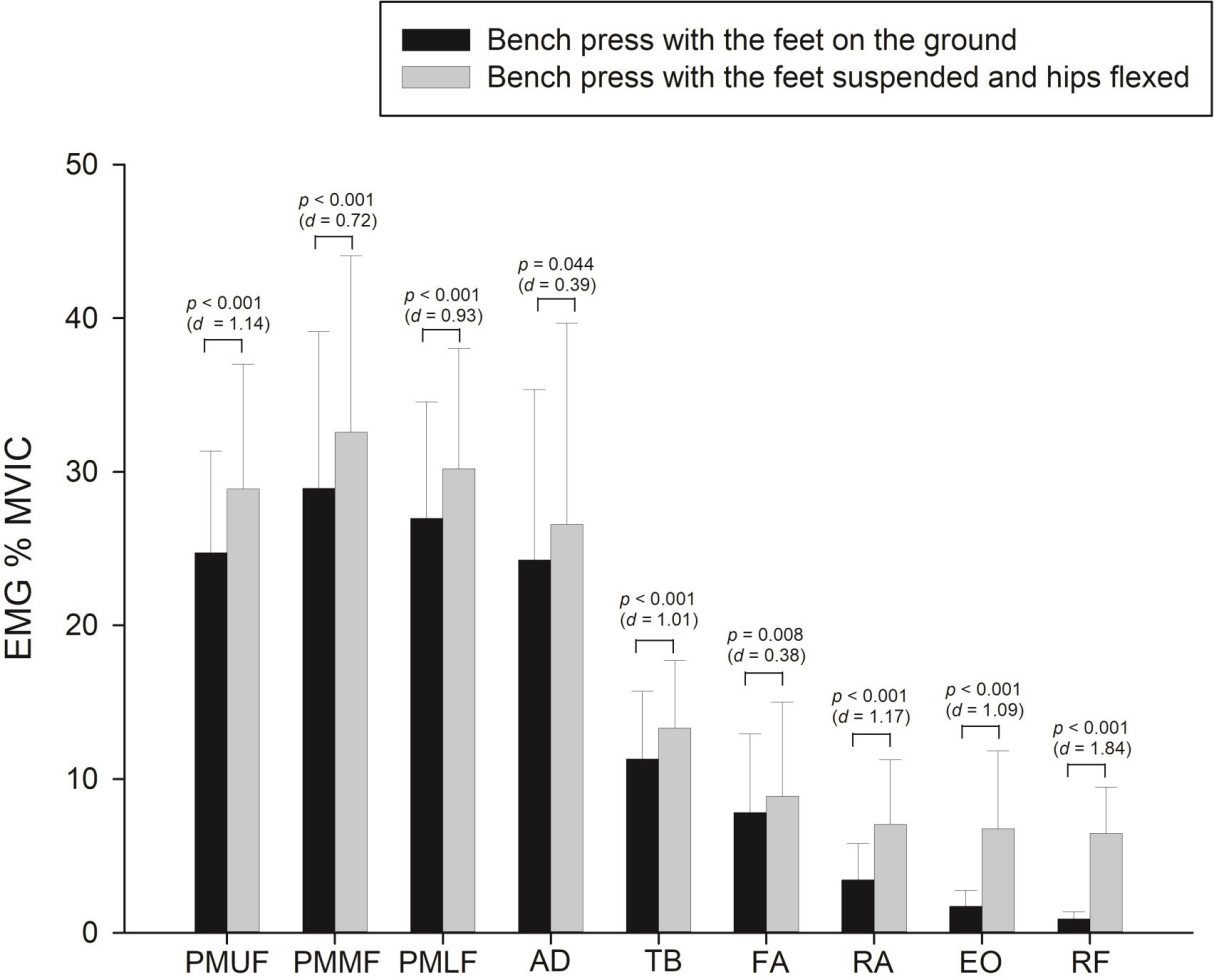
Relative muscle activity (expressed as % MVIC) of the pectoralis major upper fibers (PMUF), pectoralis major middle fibers (PMMF), pectoralis major lower fibers (PMLF), anterior deltoid (AD), triceps brachii (TB), forearm (FA), rectus abdominis (RA), external oblique (EO), and rectus femoris (RF) during bench press with the feet on the ground vs with the feet suspended and hips flexed.

During the bench press with the feet suspended and hips flexed, we observed higher activation of all evaluated muscles (*p* < 0.001; *d* > 0.7) than the AD (*p* < 0.05) and FA (*p* < 0.01) muscles, which showed a small effect size (*d *~ 0.3). The muscles that showed greater differences in activation were: RF and EO (~5%; *p* < 0.001; *d* > 1), followed by the PMUF (~4%; *p* < 0.001; *d* > 1) and PMMF, PMLF and RA (~3%; *p* < 0.001; *d* > 7). Finally, the TB showed ~1.5% higher activation (*p* 1< 0.001; *d* > 1) (Fig 3).

## Discussion

The objective of the present study was to evaluate and compare the levels of electromyographic activation in the pectoralis major (three portions), anterior deltoid, triceps brachii, forearm, rectus abdominis, external oblique, and rectus femoris muscles during a bench press in two situations: 1) with the feet on the ground and the hips in a neutral position; and 2) with active hip and knee flexion at 90°.

The main finding of this study is the significantly greater electromyographic activation of all of the evaluated muscles when the bench press exercise was performed with active hip and knee flexion at 90° both in absolute EMG values in microvolts and as percentage of the MVIC. To our knowledge, no previous studies evaluated and compared the electromyographic activation of the bench press exercise in both situations; as a result, we are unable to state whether our findings are consistent with those of other studies. However, the present study does make it clear that performing the bench press exercise with active hip and knee flexion at 90° results in significant muscle activation. In a previous study, Saeterbakken and Fimland [16] evaluated electromyographic activity in bench press on stable and unstable surfaces. These authors found that stable bench press had greater pectoralis and triceps EMG activity compared with the unstable surfaces. These results are opposite to our findings, although in that study the unstable surface was placed in the thoracic spine instead of removing the support of the feet, as in our study.

Furthermore, the pectoralis major muscle, primarily the medial fibers (sternal portion), was most activated in this exercise in both situations. This finding is consistent with those of previous studies [8, 11] since the bench press exercise is mainly performed to condition the pectoralis major muscle [6].

In the present study, after the pectoralis major, the anterior deltoid and triceps brachii showed the most electromyographic involvement in the bench press exercise. This musculature is commonly evaluated in this exercise [11, 12, 14]. However, in contrast to our study, Giorgio et al. [14] and Snyder and Fry [11] reported greater activation of the triceps brachii than the anterior deltoid muscle. This difference in the results of the two studies is possibly due to the width of the grip established in these studies. For example, Snyder and Fry [11] used a self-selected grip for each participant. It is possible that the grip was narrower than the one used in the present study (150% of the biacromial distance). In this sense, Lehman [41] have noted that in the bench press exercise, a narrower bar grip results in correspondingly greater activation of the triceps brachii. However, using a wider grip than in the present study (200% of the biacromial distance), Giorgio et al. [14], also found greater activation of the triceps brachii than of the anterior deltoid. However, the authors neither proved nor discussed this finding.

On the other hand, another result from the present study that should be highlighted is the significantly greater activation of the forearm muscles (flexor digitorum), abdominal muscles (rectus abdominis and external oblique), and rectus femoris in the bench press exercise with active hip and knee flexion at 90°. These results may be due to the greater instability generated in the exercise to be executed without the support of the feet on the ground. That is, the only support given is by the back and head on the bench. For this reason, these muscles would need to contract with greater intensity to stabilize the body while the exercise is being performed. It is noteworthy that the muscles are not a frequent topic of study in bench press research. However, in the present study, the evaluation was determined due to the position of instability that is produced by not having the support of the feet on the ground. Accordingly, several studies have evaluated the abdominal muscles when the bench press exercise is performed in an unstable situation, mainly on a Swiss ball [16–19]. Marshall and Murphy [17] observed a significant increase in muscle activity in the anterior deltoid and abdominal muscles when the bench press exercise was performed on a Swiss ball versus a stable bench. Saeterbakken and Fimland [42] noted significantly greater muscle activation of the anterior rectus abdominis when the bench press was performed on a Swiss ball versus a stable bench. Norwood et al. [18] observed significant increases in the activation of the evaluated muscles (latissimus dorsi, rectus abdominus, erector spinae, internal oblique, soleus, and biceps femoris) in three instability situations when the bench press was performed versus the stability condition. In contrast to our results and the studies referenced above, Uribe et al. [19] found no significant differences in muscle activation level when they investigated the bench press exercise in a stable vs unstable situation. It is possible, as the authors themselves noted, that the results are due to the high loads used in their study (80% 1RM). This load, together with the participants’ body weight, could deform (squish) the Swiss ball so much that it remained as flat as a stable bench [19]. On the other hand, regarding this result, we believe that when such a heavy load is being lifted, the muscle activation level is so high that it would not be possible to observe significant differences between the two conditions (stable vs unstable). Future studies should test this hypothesis.

Another key point of the present study is the significantly greater activation of the rectus femoris muscle in the bench press exercise with active hip flexion compared to the condition of the feet on the ground. Logically, this situation would be the normal one, since one of the main functions of the rectus femoris muscle is hip flexion in addition to knee extension. The issue is that the activation of the hip flexor muscles is associated with an increase in shear stress on the lumbar intervertebral discs [43]; in this situation, lumbar problems could be triggered over time [44] so much so that in abdominal strengthening exercises, it is not advisable to activate the hip flexor muscles to decrease the spinal load [45]. In addition, the recommendation is to avoid active hip and knee flexion during exercises that involve the upper limbs [46]. Therefore, considering these methodological premises, it would be possible to conclude that performing the bench press exercise with active hip and knee flexion at 90° could be discouraged due to body position instability and vertebral stress increases.

On the one hand, it should be noted that the present study exhibited a series of limitations that should be addressed in future work. For example, the speed of the movement was performed 2 second by eccentric phase and 2 second by concentric phase for obtaining a clearer EMG signal [34, 35]. It would have been interesting to evaluate this muscle activation including a maximal voluntary speed for further studies since it is frequently used nowadays in resistance training [47, 48].

On the other hand, hip flexion was performed actively. It would have been interesting to evaluate the bench press exercise with passive hip flexion (with support of the feet or heels on a stand) to minimize activation of the hip flexor muscles (rectus femoris), a case in which the dorsal support would be unstable. We could thus observe whether the greater activation of the evaluated muscles is due to the instability generated by the loss of a point of support (the feet) or was a result of increased shear stress on the lumbar spine, which produces more intense activation of the abdominal muscles in their function of protecting and stabilizing the spinal column.

## Conclusions

The bench press exercise with active hip and knee flexion at 90° significantly increased activation of the pectoralis major (clavicular portion, sternal portion, and costal portion), anterior deltoid, triceps brachii (medial head), forearm (flexor digitorum), rectus abdominis, external oblique, and rectus femoris muscles (quadriceps) muscles compared with the bench press exercise with the feet on the ground, with the same load (kg) in both positions. For this reason, to perform the bench press exercise with flexed hips could be recommended for training in sports where the upper limbs and hip flexor muscles are required. However, more studies are needed to analyze the influence of hip flexor muscle activation on lumbar spine stress in the bench press exercise.

## Supporting information

S1 Table This is the S1 Descriptive characteristics of sample. Mean (standard deviation).
(DOCX)

S2 Table This is the S1 Muscle activity (expressed as mV) of the pectoralis major (upper, middle, and lower fibers), anterior deltoid, triceps brachii, forearm, rectus abdominis, external oblique, and rectus femoris during bench press with the feet on the ground vs with the feet suspended and hips flexed. CI: confidence interval.
(DOCX)

S1 Fig This is the S1 Relative muscle activity (expressed as % MVIC) of the pectoralis major upper fibers (PMUF), pectoralis major middle fibers (PMMF), pectoralis major lower fibers (PMLF), anterior deltoid (AD), triceps brachii (TB), forearm (FA), rectus abdominis (RA), external oblique (EO), and rectus femoris (RF) during bench press with the feet on the ground vs with the feet suspended and hips flexed. (DOCX)
