# Supplementary material for: Evaluation and comparison of electromyographic activity in bench press with feet on the ground and active hip flexion
Source: PLoS One. 2019 Jun 14;14(6):e0218209. doi: 10.1371/journal.pone.0218209 (PMC6568408; doi:10.1371/journal.pone.0218209)
Supplement: S1 Table — Mean (standard deviation). (DOCX) [file pone.0218209.s001.docx]

**S1 Table. Sample characteristics**

| **Descriptive statistics** | | | | | |
| --- | --- | --- | --- | --- | --- |
|  | N | Min | Max | Mean | SD |
| Age | 20 | 19,00 | 29,00 | 22,8000 | 3,00175 |
| Body mass_kg | 20 | 62,00 | 96,00 | 77,0000 | 8,88523 |
| Height _m | 20 | 1,70 | 1,92 | 1,7960 | ,05807 |
| BMI | 20 | 20,96 | 28,37 | 23,8365 | 2,16308 |
| biacromial distance 150% cm | 20 | 64,50 | 81,00 | 69,5250 | 3,99169 |
| 1RM (kg) | 20 | 60,00 | 110,00 | 85,0000 | 12,87593 |
| Nº participants | 20 |  |  |  |  |
