# Supplementary material for: Evaluation and comparison of electromyographic activity in bench press with feet on the ground and active hip flexion
Source: PLoS One. 2019 Jun 14;14(6):e0218209. doi: 10.1371/journal.pone.0218209 (PMC6568408; doi:10.1371/journal.pone.0218209)
Supplement: S2 Table — CI: confidence interval. (DOCX) [file pone.0218209.s002.docx]

**S2 Table. T-Student % MVIC Bench press feet on the ground VS flexed hips**

| **Paired samples statistics** | | | | | |
| --- | --- | --- | --- | --- | --- |
|  | | Mean | N | SD | SD error |
| Par 1 | EMG_Pect_Upper_Press_Horz_150_Feet_Ground | 328,0000 | 20 | 119,01216 | 26,61193 |
|  | EMG_Pect_Upper_Press_Horz_150_Flex_hips | 378,3500 | 20 | 124,60011 | 27,86143 |
| Par 2 | EMG_Pect_Med_Press_Horz_150_Feet_Ground | 308,5500 | 20 | 200,78726 | 44,89740 |
|  | EMG_Pect_Med_Press_Horz_150_Flex_hips | 335,9500 | 20 | 197,51895 | 44,16658 |
| Par 3 | EMG_Pect_Inf_Press_Horz_150_Feet_Ground | 335,2000 | 20 | 152,37302 | 34,07164 |
|  | EMG_Pect_Inf_Press_Horz_150_Flex_hips | 371,6500 | 20 | 148,89215 | 33,29330 |
| Par 4 | EMG_Deltoid_Press_Horz_150_Feet_Ground | 565,6500 | 20 | 247,69915 | 55,38721 |
|  | EMG_Deltoid_Press_Horz_150_Flex_hips | 622,2000 | 20 | 288,69080 | 64,55323 |
| Par 5 | EMG_Tríceps_Press_Horz_150_Feet_Ground | 240,1000 | 20 | 88,46641 | 19,78169 |
|  | EMG_Tríceps_Press_Horz_150_Flex_hips | 267,3000 | 20 | 88,64780 | 19,82225 |
| Par 6 | EMG_Forearm_Press_Horz_150_Feet_Ground | 144,1500 | 20 | 97,06931 | 21,70536 |
|  | EMG_Forearm_Press_Horz_150_Flex_hips | 163,2500 | 20 | 114,54251 | 25,61248 |
| Par 7 | EMG_Abd_Press_Horz_150_Feet_Ground | 31,3500 | 20 | 11,15572 | 2,49449 |
|  | EMG_Abd_Press_Horz_150_Flex_hips | 70,8000 | 20 | 42,98666 | 9,61211 |
| Par 8 | EMG_Ext_Obliq_Press_Horz_150_Feet_Ground | 17,5000 | 20 | 9,20812 | 2,05900 |
|  | EMG_Ext_Obliq_Press_Horz_150_Flex_hips | 70,8500 | 20 | 57,74652 | 12,91251 |
| Par 9 | EMG_Quadriceps_Press_Horz_150_Feet_Ground | 10,4737 | 19 | 3,61122 | ,82847 |
|  | EMG_Quadriceps_Press_Horz_150_Flex_hips | 81,5263 | 19 | 40,11839 | 9,20379 |

| **Correlations** | | | | |
| --- | --- | --- | --- | --- |
|  | | N | Correlation | Sig. |
| Par 1 | EMG_Pect_Upper_Press_Horz_150_Feet_Ground & EMG_Pect_Upper_Press_Horz_150_Flex_hips | 20 | ,948 | ,000 |
| Par 2 | EMG_Pect_Med_Press_Horz_150_Feet_Ground & EMG_Pect_Med_Press_Horz_150_Flex_hips | 20 | ,987 | ,000 |
| Par 3 | EMG_Pect_Inf_Press_Horz_150_Feet_Ground & EMG_Pect_Inf_Press_Horz_150_Flex_hips | 20 | ,967 | ,000 |
| Par 4 | EMG_Deltoid_Press_Horz_150_Feet_Ground & EMG_Deltoid_Press_Horz_150_Flex_hips | 20 | ,941 | ,000 |
| Par 5 | EMG_Tríceps_Press_Horz_150_Feet_Ground & EMG_Tríceps_Press_Horz_150_Flex_hips | 20 | ,961 | ,000 |
| Par 6 | EMG_Forearm_Press_Horz_150_Feet_Ground & EMG_Forearm_Press_Horz_150_Flex_hips | 20 | ,971 | ,000 |
| Par 7 | EMG_Abd_Press_Horz_150_Feet_Ground & EMG_Abd_Press_Horz_150_Flex_hips | 20 | ,105 | ,658 |
| Par 8 | EMG_Ext_Obliq_Press_Horz_150_Feet_Ground & EMG_Ext_Obliq_Press_Horz_150_Flex_hips | 20 | ,484 | ,030 |
| Par 9 | EMG_Quadriceps_Press_Horz_150_Feet_Ground & EMG_Quadriceps_Press_Horz_150_Flex_hips | 19 | -,450 | ,053 |

| **Paired samples statistics** | | | | | | |
| --- | --- | --- | --- | --- | --- | --- |
|  | | Difference paired samples | | | | |
|  |  | Mean | SD | SD error | 95% CI difference | |
|  |  |  |  |  | Inf | Upper |
| Par 1 | EMG_Pect_Upper_Press_Horz_150_Feet_Ground - EMG_Pect_Upper_Press_Horz_150_Flex_hips | -50,35000 | 39,49987 | 8,83244 | -68,83651 | -31,86349 |
| Par 2 | EMG_Pect_Med_Press_Horz_150_Feet_Ground - EMG_Pect_Med_Press_Horz_150_Flex_hips | -27,40000 | 32,31164 | 7,22510 | -42,52231 | -12,27769 |
| Par 3 | EMG_Pect_Inf_Press_Horz_150_Feet_Ground - EMG_Pect_Inf_Press_Horz_150_Flex_hips | -36,45000 | 38,80243 | 8,67649 | -54,61010 | -18,28990 |
| Par 4 | EMG_Deltoid_Press_Horz_150_Feet_Ground - EMG_Deltoid_Press_Horz_150_Flex_hips | -56,55000 | 100,86284 | 22,55362 | -103,75526 | -9,34474 |
| Par 5 | EMG_Tríceps_Press_Horz_150_Feet_Ground - EMG_Tríceps_Press_Horz_150_Flex_hips | -27,20000 | 24,80789 | 5,54721 | -38,81045 | -15,58955 |
| Par 6 | EMG_Forearm_Press_Horz_150_Feet_Ground - EMG_Forearm_Press_Horz_150_Flex_hips | -19,10000 | 30,92078 | 6,91410 | -33,57137 | -4,62863 |
| Par 7 | EMG_Abd_Press_Horz_150_Feet_Ground - EMG_Abd_Press_Horz_150_Flex_hips | -39,45000 | 43,25746 | 9,67266 | -59,69511 | -19,20489 |
| Par 8 | EMG_Ext_Obliq_Press_Horz_150_Feet_Ground - EMG_Ext_Obliq_Press_Horz_150_Flex_hips | -53,35000 | 53,89197 | 12,05061 | -78,57222 | -28,12778 |
| Par 9 | EMG_Quadriceps_Press_Horz_150_Feet_Ground - EMG_Quadriceps_Press_Horz_150_Flex_hips | -71,05263 | 41,86814 | 9,60521 | -91,23243 | -50,87283 |

| **Paired samples statistics** | | | | |
| --- | --- | --- | --- | --- |
|  | | t | gl | Sig. (bilateral) |
|  |  |  |  |  |
|  |  |  |  |  |
| Par 1 | EMG_Pect_Upper_Press_Horz_150_Feet_Ground - EMG_Pect_Upper_Press_Horz_150_Flex_hips | -5,701 | 20 | ,000 |
| Par 2 | EMG_Pect_Med_Press_Horz_150_Feet_Ground - EMG_Pect_Med_Press_Horz_150_Flex_hips | -3,792 | 20 | ,001 |
| Par 3 | EMG_Pect_Inf_Press_Horz_150_Feet_Ground - EMG_Pect_Inf_Press_Horz_150_Flex_hips | -4,201 | 20 | ,000 |
| Par 4 | EMG_Deltoid_Press_Horz_150_Feet_Ground - EMG_Deltoid_Press_Horz_150_Flex_hips | -2,507 | 20 | ,021 |
| Par 5 | EMG_Tríceps_Press_Horz_150_Feet_Ground - EMG_Tríceps_Press_Horz_150_Flex_hips | -4,903 | 20 | ,000 |
| Par 6 | EMG_Forearm_Press_Horz_150_Feet_Ground - EMG_Forearm_Press_Horz_150_Flex_hips | -2,762 | 20 19 | ,012 |
| Par 7 | EMG_Abd_Press_Horz_150_Feet_Ground - EMG_Abd_Press_Horz_150_Flex_hips | -4,079 | 20 | ,001 |
| Par 8 | EMG_Ext_Obliq_Press_Horz_150_Feet_Ground - EMG_Ext_Obliq_Press_Horz_150_Flex_hips | -4,427 | 20 | ,000 |
| Par 9 | EMG_Quadriceps_Press_Horz_150_Feet_Ground - EMG_Quadriceps_Press_Horz_150_Flex_hips | -7,397 | 20 | ,000 |
