# Supplementary material for: Evaluation and comparison of electromyographic activity in bench press with feet on the ground and active hip flexion
Source: PLoS One. 2019 Jun 14;14(6):e0218209. doi: 10.1371/journal.pone.0218209 (PMC6568408; doi:10.1371/journal.pone.0218209)
Supplement: S1 Fig — (DOCX) [file pone.0218209.s003.docx]

**S1 Figure. T-Student % MVIC Bench press feet on the ground VS flexed hips**

| **Paired samples statistics** | | | | | |
| --- | --- | --- | --- | --- | --- |
|  | | Mean | N | SD | Mean SD error |
| Par 1 | Porc_MCV_Pectoralis_Upper_PressHorz_150_Feet_Ground | 24,7513 | 20 | 6,62745 | 1,48194 |
|  | Porc_MCV_Pect_Upper_Press_Horz_150_Flex_hips | 28,8988 | 20 | 8,12406 | 1,81660 |
| Par 2 | Porc_MCV_Pectoralis_Med_PressHorz_150_Feet_Ground | 28,9392 | 20 | 10,20879 | 2,28276 |
|  | Porc_MCV_Pect_Med_Press_Horz_150_Flex_hips | 32,5836 | 20 | 11,49093 | 2,56945 |
| Par 3 | Porc_MCV_Pectoralis_Inf_PressHorz_150_Feet_Ground | 26,9886 | 20 | 7,58718 | 1,69654 |
|  | Porc_MCV_Pect_Inf_Press_Horz_150_Flex_hips | 30,2116 | 20 | 7,80945 | 1,74625 |
| Par 4 | Porc_MCV_Deltoid_PressHorz_150_Feet_Ground | 24,2738 | 20 | 11,09908 | 2,48183 |
|  | Porc_MCV_Deltoid_Press_Horz_150_Flex_hips | 26,5637 | 20 | 13,12158 | 2,93407 |
| Par 5 | Porc_MCV_Triceps_PressHorz_150_Feet_Ground | 11,7921 | 20 | 4,06337 | ,90860 |
|  | Porc_MCV_Triceps_Press_Horz_150_Flex_hips | 13,3116 | 20 | 4,40120 | ,98414 |
| Par 6 | Porc_MCV_Forearm_PressHorz_150_Feet_Ground | 7,8317 | 20 | 5,10363 | 1,14121 |
|  | Porc_MCV_Forearm_Press_Horz_150_Flex_hips | 8,8873 | 20 | 6,13441 | 1,37170 |
| Par 7 | Porc_MCV_RectoAbdom_PressHorz_150_Feet_Ground | 3,4369 | 20 | 2,39497 | ,53553 |
|  | Porc_MCV_Recto_Abdom_Press_Horz_150_Flex_hips | 7,0538 | 20 | 4,21749 | ,94306 |
| Par 8 | Porc_MCV_OblicuoExterno_PressHorz_150_Feet_Ground | 1,7222 | 20 | 1,02069 | ,22823 |
|  | Porc_MCV_OblicuoExterno_Press_Horz_150_Flex_hips | 6,7662 | 20 | 5,06800 | 1,13324 |
| Par 9 | Porc_MCV_RectoAntQuadriceps_PressHorz_150_Feet_Ground | ,8998 | 20 | ,47443 | ,10884 |
|  | Porc_MCV_RectoAnt_Quadriceps_Press_Horz_150_Flex_hips | 6,4504 | 20 | 3,03403 | ,69605 |

| **Correlations** | | | | |
| --- | --- | --- | --- | --- |
|  | | N | Correlation | Sig. |
| Par 1 | Porc_MCV_Pectoralis_Upper_PressHorz_150_Feet_Ground & Porc_MCV_Pect_Upper_Press_Horz_150_Flex_hips | 20 | ,928 | ,000 |
| Par 2 | Porc_MCV_Pectoralis_Med_PressHorz_150_Feet_Ground & Porc_MCV_Pect_Med_Press_Horz_150_Flex_hips | 20 | ,947 | ,000 |
| Par 3 | Porc_MCV_Pectoralis_Inf_PressHorz_150_Feet_Ground & Porc_MCV_Pect_Inf_Press_Horz_150_Flex_hips | 20 | ,924 | ,000 |
| Par 4 | Porc_MCV_Deltoid_PressHorz_150_Feet_Ground & Porc_MCV_Deltoid_Press_Horz_150_Flex_hips | 20 | ,937 | ,000 |
| Par 5 | Porc_MCV_Triceps_PressHorz_150_Feet_Ground & Porc_MCV_Triceps_Press_Horz_150_Flex_hips | 20 | ,943 | ,000 |
| Par 6 | Porc_MCV_Forearm_PressHorz_150_Feet_Ground & Porc_MCV_Forearm_Press_Horz_150_Flex_hips | 20 | ,976 | ,000 |
| Par 7 | Porc_MCV_RectoAbdom_PressHorz_150_Feet_Ground & Porc_MCV_Recto_Abdom_Press_Horz_150_Flex_hips | 20 | ,693 | ,001 |
| Par 8 | Porc_MCV_OblicuoExterno_PressHorz_150_Feet_Ground & Porc_MCV_OblicuoExterno_Press_Horz_150_Flex_hips | 20 | ,535 | ,015 |
| Par 9 | Porc_MCV_RectoAntQuadriceps_PressHorz_150_Feet_Ground & Porc_MCV_RectoAnt_Quadriceps_Press_Horz_150_Flex_hips | 20 | ,118 | ,630 |

| Paired samples statistics | | | | | | | | | |
| --- | --- | --- | --- | --- | --- | --- | --- | --- | --- |
|  | | Differences | | | | | t | gl | Sig. (bilateral) |
|  |  | Mean | SD | Mean SD error | 95% CI difference | |  |  |  |
|  |  |  |  |  | Inf | Upper |  |  |  |
| Par 1 | Porc_MCV_Pectoralis_Upper_PressHorz_150_Feet_Ground - Porc_MCV_Pect_Upper_Press_Horz_150_Flex_hips | -4,14752 | 3,15853 | ,70627 | -5,62576 | -2,66929 | -5,872 | 20 | ,000 |
| Par 2 | Porc_MCV_Pectoralis_Med_PressHorz_150_Feet_Ground - Porc_MCV_Pect_Med_Press_Horz_150_Flex_hips | -3,64444 | 3,74181 | ,83670 | -5,39567 | -1,89322 | -4,356 | 20 | ,000 |
| Par 3 | Porc_MCV_Pectoralis_Inf_PressHorz_150_Feet_Ground - Porc_MCV_Pect_Inf_Press_Horz_150_Flex_hips | -3,22305 | 3,00096 | ,67103 | -4,62754 | -1,81856 | -4,803 | 20 | ,000 |
| Par 4 | Porc_MCV_Deltoid_PressHorz_150_Feet_Ground - Porc_MCV_Deltoid_Press_Horz_150_Flex_hips | -2,28989 | 4,74399 | 1,06079 | -4,51015 | -,06964 | -2,159 | 20 | ,044 |
| Par 5 | Porc_MCV_Triceps_PressHorz_150_Feet_Ground - Porc_MCV_Triceps_Press_Horz_150_Flex_hips | -1,51950 | 1,46427 | ,32742 | -2,20480 | -,83420 | -4,641 | 20 | ,000 |
| Par 6 | Porc_MCV_Forearm_PressHorz_150_Feet_Ground - Porc_MCV_Forearm_Press_Horz_150_Flex_hips | -1,05550 | 1,59499 | ,35665 | -1,80198 | -,30902 | -2,959 | 20 | ,008 |
| Par 7 | Porc_MCV_RectoAbdom_PressHorz_150_Feet_Ground - Porc_MCV_Recto_Abdom_Press_Horz_150_Flex_hips | -3,61690 | 3,08590 | ,69003 | -5,06114 | -2,17265 | -5,242 | 20 | ,000 |
| Par 8 | Porc_MCV_OblicuoExterno_PressHorz_150_Feet_Ground - Porc_MCV_OblicuoExterno_Press_Horz_150_Flex_hips | -5,04396 | 4,60322 | 1,02931 | -7,19834 | -2,88959 | -4,900 | 20 | ,000 |
| Par 9 | Porc_MCV_RectoAntQuadriceps_PressHorz_150_Feet_Ground - Porc_MCV_RectoAnt_Quadriceps_Press_Horz_150_Flex_hips | -5,55056 | 3,01503 | ,69170 | -7,00376 | -4,09736 | -8,025 | 20 | ,000 |
